# Exercise and education for community-dwelling older participants with knee osteoarthritis: a video-linked programme protocol based on a randomised controlled trial

**DOI:** 10.1186/s12891-021-04331-4

**Published:** 2021-05-22

**Authors:** Lin Wang, Suhang Xie, Tianjie Bao, Siyi Zhu, Qiu Liang, Xiaoyi Wang, Ruishi Zhang, Xiaona Xiang, Chunping Du, Chengqi He

**Affiliations:** 1 Department of Rehabilitation Medicine, West China Hospital, Sichuan University, No. 37 Guoxue Xiang, Chengdu, Sichuan 610041 P. R. China; 2 Key Laboratory of Rehabilitation Medicine, West China Hospital, Sichuan University, Chengdu, Sichuan 610041 P. R. China; 3 Institute of Rehabilitation Medicine, West China Hospital, Sichuan University, Chengdu, Sichuan 610041 P. R. China

**Keywords:** Community-dwelling older adults, Knee osteoarthritis, Exercise, Education, Irritability, Psychosocial mechanism

## Abstract

**Background:**

Neuromuscular and quadriceps exercises have been shown to be effective approaches to relieve pain and to improve function for patients with knee osteoarthritis. In this study, we aim to provide an informative feasible model in which therapeutic exercise and education will be undertaken with physiotherapy supervision and instruction via video link. We also aim to explore the relationship between program-induced pain alleviation/functional improvements and reduction in irritability, which might be mediated through program-induced psychosocial benefits.

**Methods:**

In this proposed two-parallel group (neuromuscular exercise versus quadriceps exercise), single-blinded, randomised controlled trial, participants aged ≥50 years with osteoarthritic knee pain will undergo a 12-week intervention, comprising video-linked education, supervised exercises, and a 12-week follow-up. Seven measurements will be taken to collect longitudinal data. A generalised estimating equation will be used to establish the adjusted difference in effectiveness on pain, function, irritability, and psychosocial outcomes between participants undertaking neuromuscular exercises and those undertaking quadriceps exercises. The primary outcomes are overall average pain in the knee joint during walking, as assessed through the 11-point Numerical Pain Rating Scale, and the Western Ontario and McMaster Universities osteoarthritis index physical function subscale. Furthermore, pressure pain threshold and changes in self-report pain scores pre-, during, and post-exercise were also measured as an indication of irritability. In addition, both the 6-min walk test and a timed up & go test were used to assess walking function performance. Finally, patients’ emotions (e.g., fear and catastrophising), self-trust, needs in terms of disease knowledge, mental resilience, social support and health-related quality of life were investigated. Two four-wave cross-lagged models will be used to investigate directional relationships, aiming to investigate the complex mechanisms concerning the effects of exercise programmes.

**Discussion:**

Through summarising the study’s strengths and limitations, this study may provide promising insights in terms of exercise therapy optimisation for people with knee osteoarthritis and/or other chronic pain within a psychosocial framework.

**Trial registration:**

ChiCTR2100041978 (chictr.org.cn), January 10, 2021.

## Background

Knee osteoarthritis (KOA) was reported to have involved living a global total of 8.3 million years with a disability between 1997 and 2017 [1], and 4.15 million years lived with a disability between 2012 and 2018 in China [2]. With global ageing and an increased prevalence in obesity [3], a continued rise is anticipated. Clinical practice guidelines strongly recommend education and individualised exercise as a first-line option for managing KOA symptoms [4, 5].

Neuromuscular exercise, based on biomechanics and neuromuscular control principles, comprises a series of motions or actions aimed at improving sensorimotor control and achieving functional stability [6], in which there is an emphasis on the quality of open and closed chain movements through performing functionally weight-bearing exercises with correct posture and alignment [7]. Quadriceps exercises aim to improve muscular mass, strength, and endurance through undertaking a range of reduced weight-bearing open-chain exercises to limit direct pressure on the knee, to strengthen the major muscle groups, and to improve joint stability [8].

Recent clinical trials have suggested that both exercise regimens could markedly ease pain and improve function [9–11]; however, whether significant differences in pain-relieving and functional improvement effects exist between the two exercise types is controversial. There is, however, limited evidence supporting the difference in effects between these two types of exercise when they are combined with education and when they are delivered using mobile online programs and physiotherapy supervision [12].

Peripheral and central pain sensitisation is considered to be the cause of KOA-related pain, the features of which are associated with self-reported pain severity [13, 14]. In quantitative sensory testing, pain sensitisation is often expressed as a pressure pain threshold (PPT) and temporal summation due to mechanical stimulation [15]; whereas, in clinical pain management, physiotherapists focus on irritability [16]. According to Maitland’s definition, the more severe the pain is in response to a given physical activity or mechanical stimulation, the more irritable the pain becomes. Irritability can be assessed as the intensity of activity required to cause pain, severity of induced pain, and duration of pain [17].

Pain sensitivity during a given physical activity has been reported to be an independent predictor of activity performance and pain-related impairment in communal participation for patients with KOA [16]. A recent trial that measured the trajectory of pain during 12 weeks of neuromuscular training reported that changes in pain during training, induced through progressive exercise, decreased over time and reached a plateau in the 10th week [18]. Moreover, it has been shown that exercise-induced analgesia is characterised as reduced sensitivity to painful stimuli post-training [19]. As indicated, assessing irritability and the long- or short-term effects of exercise programs in relieving pain could be relevant [20].

Psychological effects of exercise include acquiring illness knowledge, reducing helplessness, increasing self-efficacy (a person’s self-trust in performing certain goal-oriented tasks [21]), optimising coping strategies (the efforts to deal with and minimise the effects of illness [22]), strengthening social support, and relieving depression [23]. Moreover, KOA symptoms, especially chronic pain and functional restrictions [3, 24], affect patient psychosocial status. Exactly, exercise has benefits within psychosocial domains that vary with the effects of controlled symptoms or irritability, rather than remaining static and unchanging characteristics [23, 25]. Changes in mental characteristics could be in reaction to analgesic and functional benefits and other behaviours involved in self-management. Self-efficacy, strengthened through KOA-specific exercise, has been reported to be an independent predictor of better functional performance [26].

In studying the association between self-efficacy and physiological efficacy, the assessment factors of psychological outcomes should be distinguished as static characteristics [27] or as a dynamically assessed progress during the exercise [26, 28], which conforms to the self-efficacy theory of Bandura [21, 29]. Besides, three out of ten women with knee osteoarthritis tend to suffer from depression [30], which could be reduced by exercise programs [31, 32]. However, as with other psychological benefits, the relationship with patient-perceived relieved pain and improved function has not yet been established.

A previous study by our research group indicated that Internet-based rehabilitation programs could improve pain but not physical function in patients with KOA [33]. In another unpublished study, we suggested that exercise effectively improved some domains of psychological health and that the management of pain and disability could be enhanced by improved psychological status (Study registration: PROSPERO 2021 CRD42021217822).

In the present study, we propose an informative feasible model of exercise for KOA. Specifically, the model involves physical therapists delivering remote video guidance for exercise and demand-oriented disease education online, as well as family support. This model may be beneficial to patients with KOA as a means to improve pain, physical function, mental health and social support. Given that a decrease in irritation could play a vital role in the improvement of pain and function [15, 17]. Psychosocial factors may also play an intermediary role in the process [23].

### Objective

This clinical trial will investigate effects on pain, function, mental health, social participation/support, and health-related quality of life for community-dwelling older participants with KOA through a 12-week program that combines progressive exercise and systematic education under the video-linked instruction of a physiotherapist and with the support of a participant’s family. The key mechanisms of irritability, physiological outcomes, mental health, and social participation/support will be further explored. We hypothesised that: (i) progressive exercise and systematic education will benefit participants with KOA, and that neuromuscular exercise will have a better effect than quadriceps exercise; (ii) pain relief benefits from a decrease in peripheral hyperalgesia after a period of regular exercise training, which is mediated through the improvement of mental health and social participation/support, and; (iii) after a period of regular exercise training, a decrease in exercise-related irritability will enhance functional improvement, which is mediated through improvement in mental health and social participation/support.

### Trial design

The design of this trial, involving video-linked instruction by physiotherapists and support from a participant’s family in relation to education combing neuromuscular exercise versus quadriceps exercise for community-dwelling older participants with KOA, is a two-armed, randomised, positive-controlled, investigator-blinded, prospective and longitudinal study with a 12-week intervention along with a further 12-week follow-up period.

## Methods

**Fig. 1:**
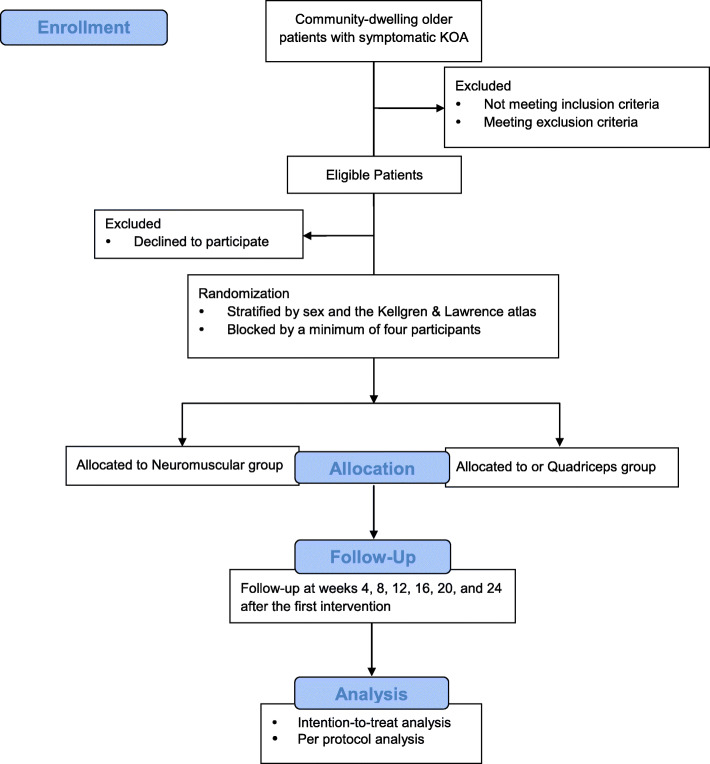
Flow diagram explaining the study protocol timeline

This protocol is guided by the Standard Protocol Items: Recommendations for Interventional Trials statement [34]. A flow diagram explaining the protocol timeline is shown in Fig. 1. The Ethics Committee on Biomedical Research, West China Hospital of Sichuan University has approved this trial (identifier: 2020–945).

### Study setting

This trial will be conducted among community-dwelling older participants with KOA in Chengdu, Sichuan Province from December 2020 to December 2021. The intervention and evaluation site will be a participant’s home or community.

### Participants

Community-dwelling older adults are potentially eligible if they: (i) are aged ≥50 years at the time of enrolment; (ii) meet the criteria for symptomatic KOA according to the American College of Rheumatology [35]; (iii) have a plain knee radiographic image indicating KOA grades 0-III, using the Kellgren & Lawrence atlas [36]; (iv) have a plain knee radiographic image showing joint space narrowing (grades 0–2), based on the Osteoarthritis Research Society International classification [36]; and (v) have the support of family members during every exercise session to ensure basic safety, the use of a video connection, and video-viewing facilities.

In relation to potential participants, exclusion criteria comprise: (i) having undergone arthroplasty, arthroscopy, or open surgery on the knee in the past 12 months, or planning to undergo an elective knee joint replacement surgery within the next 6 months; (ii) having other joint pathologies (for example, rheumatoid arthritis, severe osteoporosis, or fracture); (iii) having confirmed or suspected diseases that may restrict exercise (for example, cerebral haemangioma, exertional angina pectoris, severe anaemia, fixed-rate pacemaker, or complete atrioventricular block); (iv) being at risk of participating in exercise without supervision or screening while using the physical activity readiness questionnaire [37]; (v) having hypertension and diabetes mellitus without monitoring and treatment; (vi) having cognitive impairment as screened using Mini-Cog [38]; or (vii) having confirmed serious psychiatric disorders (for example, schizophrenia, major depression, paranoid psychosis, mania, or mental disorders due to mental retardation).

### Intervention

The 12-week intervention will comprise exercise and education sessions conducted by four certified physiotherapists experienced in managing chronic musculoskeletal pain (QL, XW, RZ, and TW). Exercise training programs will differ between two groups, that is, one group performing neuromuscular exercise versus another group performing quadriceps exercise, with education based on individual needs following the same design between the two groups. Physiotherapists will attend a 4-h standardised training before the formal start of the trial, to ensure reliability and repeatability of the delivered interventions (both education and exercise). With the implementation of the proposal, a technical partner (BC) will concurrently maintain and improve the functionality of a mobile online platform, namely, Joint Consultation, which has been developed to remind participants and deliver videos of specific home-based exercises, after which exercise diaries will be completed. In brief, reminders will be sent electronically concerning the video links and self-exercise for participants to accept the exercise prescription, to view videos of the exercises (also on the online platform), and to complete the exercise diary on Joint Consultation. Furthermore, a free, open-source software program (Tencent Meeting) will be used for video links between participants and physiotherapists.

### Exercise

During the program, a participant will conduct three 30–40 min exercise sessions per week. A physiotherapist will supervise participants and deliver progressive exercise through a video connection weekly (12 sessions in total), and participants will exercise at home twice a week according to the exercise prescription (24 sessions in total). Family members will accompany the participants during each exercise session (including during video connection with the physiotherapist) to ensure safety and to provide support.

Our quadriceps exercise program will include non-weight-bearing exercises aimed to improve knee joint stability through enhancing endurance and the strength of the major muscle groups [8]. Our neuromuscular exercise program will include functional weight-bearing exercises aimed to promote functional stability along with alignment through improving sensorimotor control [6].

The messages twice a week used to remind to exercise before exercise at home, messages weekly used to remind to video-link with a physiotherapist, and exercise diary three times a week after every exercise session will be delivered to the participants with Joint Consultation. The results filling in the exercise diary feedback to the physiotherapist and clinical research assistant will be used as the record of participant attendance at-home exercise and feedback on symptom responses for calculating and monitoring adherence. Besides, the weekly video-linked session with the physiotherapist ensures the problems related to exercise and symptom responses can be informed and addressed in time, which could be considered as another action to monitor potential drop-out situations or the loss of follow-up, and reduce the number of subjects who violate the allocation plan.

The exercise testing plan and exercise prescription are based on findings from previous studies [39–41]. In detail, the neuromuscular exercises will progress in difficulty monthly, and the quadriceps exercises will progress in resistance and in the number of sets weekly from the third training, which will be determined according to a participant’s pain (defined as an average pain score ≥ 4, on an 11-point numerical pain rating scale (NPRS) [42] during exercise) and perceived exertion (defined as an average exertion between 5 and 8, on an 11-point modified rating perceived exertion scale [43] during exercise).

### Education

Participant needs-based education lasting 15–20 min will be conducted by the physiotherapist monthly through a video link (four sessions in total). The educational needs assessment tool (ENAT) [44] will be used to determine a participant’s level of interest in terms of KOA knowledge, according to which individualised education will be planned and delivered. The education content corresponding to 39 ENAT items will be guided by clinical guidelines and previous evidence, and will mainly include information on the current condition and possible outcomes for each participant, the vital role of exercise and healthy lifestyle (e.g. weight control), options and methods to help alleviate symptoms, and other content intended to facilitate participants’ understanding [4, 5, 45–53]. Moreover, the physiotherapists will encourage participants to review the education materials and will respond to any participants’ questions [49].

### Outcome measures

Table 1 summarises the primary and secondary outcome measures. Longitudinal evaluation will be conducted seven times in total over a 6-month period. As shown in the participant timeline (Table 2), outcome data will be collected at baseline, and at 4, 8, and 12 weeks after allocation, mostly comprising self-reported scales. Over a 3-month follow-up, evaluations will be conducted at 4, 8, and 12 weeks post-intervention.

**Table 1:** A summary of the outcome measures

Measurements	Metric of measurement	Method of measurement	Time point of primary interest
**Primary outcomes**			
Self-reported pain	0–10 (higher scores indicate more serious pain)	11-point numerical pain rating scale	Baseline, and 4, 8, 12, 16, 20, and 24 weeks after allocation
Self-reported function	0–68 (higher scores indicate more serious dysfunction)	Western Ontario and McMaster Universities osteoarthritis index (WOMAC) – physical function subscale	
**Secondary outcomes**			
Self-reported pain	0–20 (higher scores indicate more serious pain)	WOMAC pain subscale	Baseline, and 4, 8, 12, 16, 20, and 24 weeks after allocation
Functional performance	Metres (longer distance indicates better function)	6-min walk test (6MWT)	
	Seconds (shorter time indicates better function)	Timed up & go (TUG) test	
Sensitisation in resting state	Kilogram (lighter pressure indicates a greater degree of sensitisation or increased pain sensitivity)	Pressure pain threshold (PPT)	
Exercise-dependent sensitisation	0–11 (higher scores indicate a greater degree of sensitisation or increased pain sensitivity)	Changes in self-reported pain pre-, post- and during exercise via 11-point numerical pain rating scale in exercise diaries	
Catastrophising	0–52 (higher scores indicate a greater degree of pain catastrophising)	Pain catastrophising scale (PCS)	
Fear related to movements	17–68 (higher scores indicate more serious fear)	Tampa scale for kinesiophobia (TSK)	
Self-trust in exercise	0–70 (higher scores indicate stronger confidence)	Self-efficacy for exercise scale (SEES)	
Self-trust in pain	0–50 (higher scores indicate stronger confidence)	Pain self-efficacy questionnaire (PSEQ)	
Acquisition of knowledge about KOA	0–156 (higher scores indicate less knowledge of KOA)	Educational needs assessment tool (ENAT)	
Mental resilience	10–50 (higher scores indicate stronger adaptation competence in the face of problems)	10-item Connor-Davidson resilience scale (CD-RISC-10)	
Perceived social support	8–50 (higher scores indicate more satisfying social support)	Satisfaction with received social support (SRSS)	
Pain affecting social participation	16–96 (higher scores indicate more serious impairment in social participation)	Pain inference subscale of the West Haven Yale multidimensional pain inventory (WHYMPI)	
Quality of life	Standardised scores for every section (higher scores indicate more serious impairment in social participation)	36-item short-form health survey (SF-36)	Baseline, and 12 and 24 weeks after allocation
**Other assessments**			
Comorbid conditions	1–41 (higher scores indicate more complex and dangerous comorbid conditions)	Charlson comorbidity index (CCI)	Baseline
Level of physical activity	Four levels divided into inactive, light-, moderate-, hard-, and very hard-intensity.	Stanford brief activity survey (SBAS)	
Overall mental health	14–70 (higher scores indicate better psychological health)	Warwick-Edinburgh mental well-being scale (WEMWBS)	
Drugs, physical therapies, and other therapies used	Type, frequency, duration	Self-report	

*Abbreviations*: *CCI* Charlson comorbidity index, *CD-RISC-10* 10-item Connor-Davidson resilience scale, *ENAT* Educational needs assessment tool, *KOA* Knee osteoarthritis, *PCS* Pain catastrophising scale, *PPT* Pressure pain threshold, *PSEQ* Pain self-efficacy questionnaire, *SBAS* Stanford brief activity survey, *SF-36* 36-item short form health survey, *SEES* Self-efficacy for exercise scale, *SRSS* Satisfaction with received social support, *TSK* Tampa scale for kinesiophobia, *TUG* Timed up and go test, *WEMWBS* Warwick-Edinburgh mental well-being scale, *WHYMPI* West Haven Yale multidimensional pain inventory, *6MWT* Six-minute walk test


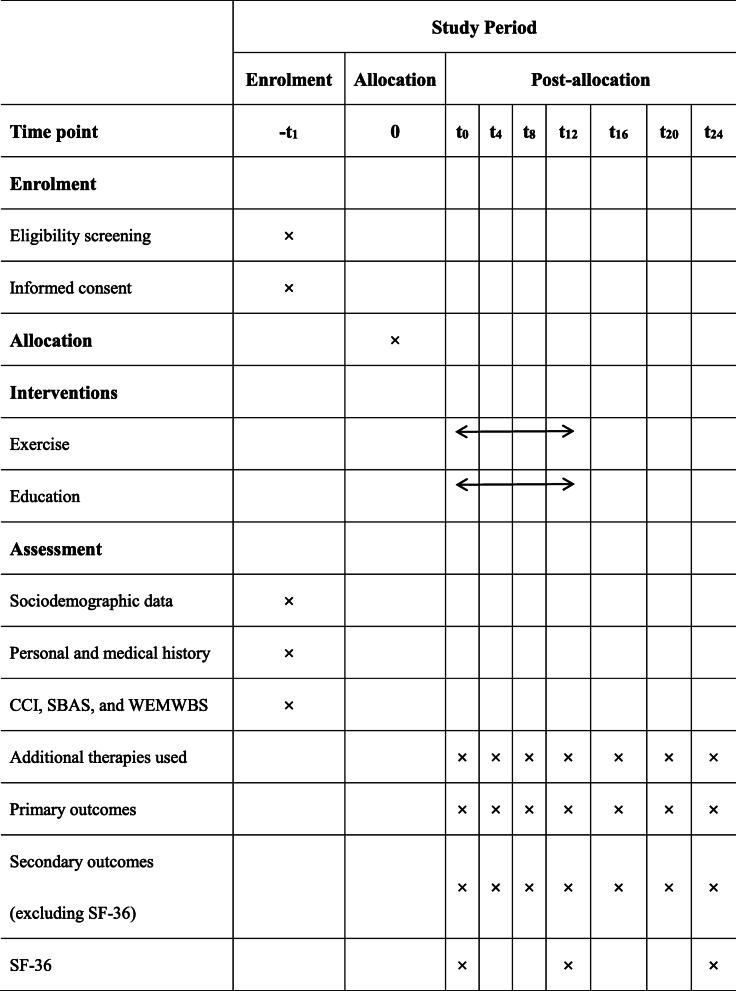

**Table 2:**
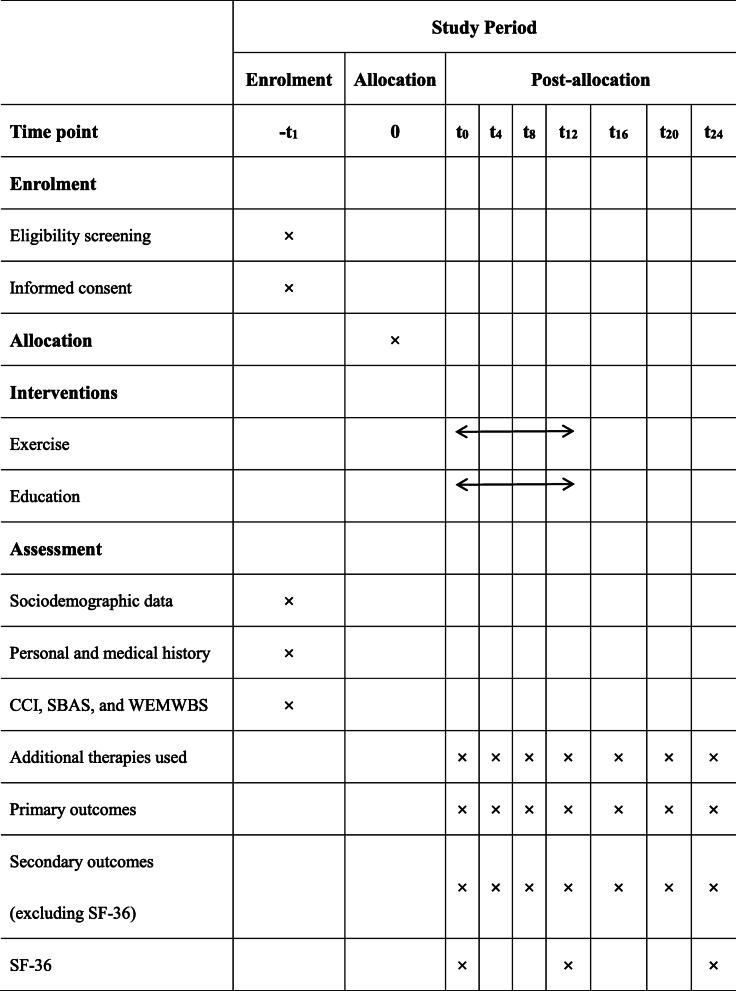
The participant timeline

*N* = week(s)

*Abbreviations*: *SF-36* 36-item short-form health survey, *CCI* Charlson comorbidity index, *SBAS* Stanford brief activity survey, *WEMWBS* Warwick-Edinburgh mental well-being scale

### Primary outcomes

Primary outcomes are as follows: (i) overall average pain in the knee joint during walking over the previous month: an 11-point NPRS will be used to investigate participant-reported pain ranging from 0 (no pain) to 10 (the worst pain), with 1.8 units having been reported as a minimum clinically important difference (MCID) [54]; and (ii) difficulty with physical function: the Western Ontario and McMaster Universities osteoarthritis index (WOMAC) physical function subscale [55] will be used to evaluate self-reported dysfunction using 17 questions. Total subscale scores will range from 0 to 68, with higher scores indicating more serious dysfunction. An MCID in terms of physical function in KOA has been determined as six units [56].

### Secondary outcomes

Secondary outcomes are as follows:

1. Knee pain over the previous 48 h, determined using the WOMAC pain subscale [55] to evaluate self-reported pain in response to five questions. Total subscale scores will range from 0 to 20, with higher scores indicating more serious pain.
2. Irritability, with PPT assessed using a handheld pressure algometer (Wagner Force Ten, FDX 25, Greenwich) to reflect peripheral and central sensitisation in a resting state. PPT is calculated through an averaging of three repeat measurements at every assessment site, namely, the upper trapezius, biceps brachii, extensor carpi radialis longus, first dorsal interosseous, rectus femoris, vastus lateralis, and tibialis anterior muscles, and the medial compartment of the knee [20, 57]. A 1 cm^2^ rubber tip press at a rate of 0.5 kg/s on the centre of the site will be used and the number recorded when pressure sensations first change to slight pain. Data concerning changes in self-reported pain via an 11-point NPRS will be extracted from exercise diaries indicating exercise-dependent peripheral sensitisation, where participants will be asked to self-report their pain scores pre- and post-exercise, and indicate their highest pain score during exercise [18].
3. Functional physical performance, determined using a 6-min walk test and a timed up & go (TUG) test to assess walking function performance [58].
4. Outcomes of mental health over the previous month, determined using the pain catastrophising scale [59], Tampa scale for kinesiophobia [60], self-efficacy for exercise scale [61], pain self-efficacy questionnaire, ENAT [44], and 10-item Connor-Davidson resilience scale [62] through evaluating the psychological status of the participants during exercise and follow-up, in terms of emotion (fear, catastrophising), self-trust, needs in terms of disease knowledge, and mental resilience domains.
5. Outcomes of social participation and social support over the previous month, determined using the pain interference subscale of the West Haven Yale multidimensional pain inventory [63], and satisfaction with received social support questionnaire [64, 65] through evaluating the psychological status of the participants during exercise and follow-up, in terms of the effects of KOA symptoms and perceived support from family, community, and society.
6. Outcomes of quality of life over the previous 3 months, determined in relation to health-related quality of life using the 36-item short-form health survey [66].

For baseline measures, the Charlson comorbidity index will be calculated to determine participants’ comordibities [67]. The Stanford brief activity survey will be used to assess participants’ baseline physical activity levels [68]. The Warwick-Edinburgh mental well-being scale will be used to assess participants’ overall mental health [69]. To determine any additional therapies used by a participant during the intervention and follow-up period, we will record drug types and dosage, physical therapy, and any other therapies.

### Sample size

PASS 15.0 (East Kaysville, USA) software will be used to test for two means in a repeated measures design, based on linear-mixed effect models [70–72], with estimations of the correlation coefficient between different observations made on the same participant set at 0.6 [9]. The sample size required will be sufficient to show a moderate effect size, that is, a 0.5 standard deviation in the between-group difference in relation to the primary outcomes (knee pain during walking and WOMAC function). With a yielding power of 80% and a significance level of 0.05, the sample size is intended to comprise 44 participants in each group. With allowance for a dropout rate of 20%, each group requires 55 enrolled participants.

### Recruitment

Dissemination of recruitment information will be transmitted by facilities or institutions providing multiple services within a certain region, including sub-district offices, primary and secondary schools (for students’ grandparents), colleges for senior students, activity centres or day-care centres for older adults in the community, and community medical institutions. Contact details or addresses concerning these facilities or institutions will be obtained from the Chengdu Civil Affairs Bureau (cdmzj.chengdu.gov.cn), Chengdu Education Bureau (edu.chengdu.gov.cn), and Chengdu Health Committee (cdwjw.chengdu.gov.cn), and these units will be invited to disseminate recruitment information on- and offline. Interested older adults will be able to voluntarily enrol online. Eligible participants will receive electronic material concerning information necessary for participation, complete the online questionnaires, and upload their signatures, with participants able to contact the research team via phone to ask questions or to consult with them.

### Participant assignment for the intervention

Eligible participants will be randomly assigned to either a neuromuscular group or a quadriceps group at a ratio of 1:1. Eligible participants will be stratified according to sex and the Kellgren & Lawrence atlas, and allocation will be computer-generated in varied blocks involving a minimum of four participants, according to the enrolment sequence per week, using the R Software 3.6.1 (R Foundation for Statistical Computing, Vienna, Austria) statistical package. Randomisation will be conducted by an independent analyst (YK) at the clinical medical school who is not involved in the study or the intervention. Participants and physiotherapists will be kept informed through online messages. Considering the nature of the intervention, an informed allocation is essential for the participants and interventionists in the study; however, evaluators, data managers, and statistical analysts will be blinded.

### Data collection and management

All outcome data will be collected by three specific evaluators (HT, ZZ, and KZ) at participants’ homes or communities and will be stored electronically. An electronic database has been developed to manage clinical data in our department [73]. Exercise diaries will be stored after every session or home-based exercise in the database attached to Joint Consolation, from which data concerning exercise-dependent changes in pain will be extracted. Our evaluators will enter the data, and data verification, mainly in relation to missing and inconsistent data, will be performed by an independent medical school graduate (LX).

### Statistical methods

We will mainly follow the intention-to-treat principle. A per-protocol analysis will be conducted among those who complete 8 of 12 in-person video-link sessions and 16 of 24 home exercises, according to allocation.

For continuous data, quantile-quantile plots will be constructed to determine the standardised residuals and histograms with normal distribution curves. For descriptive statistics, continuous data with normal distributions, continuous data with non-normal distributions, and categorical data will be described using means ± standard deviations, medians (interquartile ranges), and frequencies, respectively. The significance level will be set at 0.05 for all analyses, and a two-sided 95% confidence interval will be used.

A generalised estimating equation (GEE) will be used to test the hypothesis comparing between-group differences in terms of effects on primary and secondary outcomes. Covariates and possible confounders will be adjusted, including sociodemographic variables, baseline values of the outcome variables, stratification factors (grade of Kellgren & Lawrence atlas), and non-comparable variables at baseline [74]. Additionally, we will identify other underlying predictors from the general KOA status and review treatments that accompany our intervention or follow-up, which will be selected (*p* < 0.2) according to a univariate analysis using a backward stepwise procedure. Furthermore, for ordinal categorical secondary outcomes, we will fit the GEE using an alternating logistic regression model, assuming an exchangeable correlation structure, adjusted for all the predictors, and with final results reported as odds ratios. Given a close relation to the quasi-likelihood resolution of GEE [75], maximum likelihood estimation will be used to impute missing data.

To test the hypothesis concerning the mechanisms associated with the effects on pain and function, a cross-lagged model fitted within the structural equation modelling (SEM) will be used. SEM is an approach that allows simultaneous modelling of several variables and enables an investigation of more complex mediation models [76–78]. The variables of interest as mediators in the relationship between effects on pain/function and irritability are changes in outcomes on mental health and on social participation/support, which will be studied using two separate four-wave cross-lagged path models (during the intervention and follow-up). The time lag between each measurement will be 4 weeks. In addition to estimating cross-lagged effects, the specified model will include correlations within time points, autoregressive effects (stability), and underlying predictors, similar to the process of GEE analysis [79]. Moreover, full-information maximum likelihood will be used to handle missing data and estimate covariance matrices [80]. Additionally, data from both groups will be included in the cross-lagged model coded as two classified labels.

### Monitoring

#### Data monitoring

Not applicable.

#### Harms

Any negative health events occurring during the trial (from enrolment to the last follow-up) will be considered as adverse events, regardless of the causal relationship with the intervention. Adverse events will be identified through three mechanisms: (i) supervised care and support from the family, who will be required to accompany a participant during three exercise sessions per week (including the video-linked session with the physiotherapist); (ii) participants who report the absence of supporting family for whom the medical institution in the community will be contacted to provide supervision; and (iii) participants being required to report any adverse events to the clinical coordinator (KS) by phone or to the physiotherapist via video link. A reported adverse event will be discussed by four doctors (HG, QW, RA, and YL) at our department, and suggestions will be processed within the community medical institution or within our department, according to the suggestions, as necessary.

#### Auditing

Not applicable.

## Discussion

Considering the important role of supervision and instruction from physiotherapists in exercise therapy as well as safety during exercise and access and availability to mobile devices, we have designed and proposed to verify an intervention model that combines video-linked sessions instructed by a physiotherapist and support from family members. We proposed in this study three hypotheses to be verified in future research. Furthermore, the benefits of the informative feasible model of Internet-based exercise guidance for patients with KOA could be illustrated and, to some extent, the underlying mechanisms of the benefits could be explored.

Our study has some strengths. First, we will design an individualised exercise and education program for community-dwelling older participants. We will use online video-linked instruction and a mobile online platform to deliver the intervention, and the process will be supported by family members. ENAT results will help to guide the systematic education provided, which is intended to help achieve needs-based education.

Second, perceived psychosocial variables, such as potential mediators, will be included and assessed, thus providing greater depth to the study’s interpretation of effects in relation to pain reduction and functional improvement [76, 77]. Psychological benefits of exercise for patients with knee osteoarthritis have been well-documented by recent studies [31, 32]. However, there is a knowledge gap to bridge in the relationship between the improved interested psychological domains and the ameliorated results of pain and function before and after the exercise intervention, instead of considering the psychological assessments as a baseline or as characteristics of the participants [81–83].

Third, longitudinal data from this randomised trial using a GEE will allow us to draw conclusions with a high level of external validity [84] and will help identify the most effective form of physiotherapist-instructed and family-supported exercise through the mobile online platform. The conclusions of this study are likely to help development of an evidence-based online exercise program to manage the symptoms and mental status of patients with KOA, which will also allow optimisation of exercise therapy from a psychosocial view [4, 5, 85, 86].

However, some potential limitations also exist in the proposed study. First, self-reported heterogeneous symptoms of KOA cover a wide range of pain severity, disability, and personal experience (to address this wide range, we will assess and control psychological resilience) [87, 88]. Second, we will include variables related to changes in osteoarthritic pain during the exercise sessions based on findings from previous studies that may not have considered all the variables involved in the multidimensional nature of pain [78]. Third, the intervention in this study involved the support from the participants’ families, which only will be recorded as the number of accompanied exercise sessions by monthly survey, but not details of family support and companionship shown. Although, the details could be questioned in the interview after the period of the intervention, the evidence-based quantitative tools to assess this factor could be a future direction of study. Finally, as with all cross-lagged models, our findings concerning the mechanisms for pain are exploratory and require external validation [76–78].

### Study status

Seventy-one participants were enrolled in the study as of April 21, 2021. The enrolment of the first participant was on February 23, 2021.

## Data Availability

Enquired in advance, the corresponding author will provide the datasets of the study for rational purposes.

## Abbreviations

ENAT: Educational needs assessment tool
GEE: Generalised estimating equation
KOA: Knee osteoarthritis
MCID: Minimum clinically important difference
NPRS: Numerical pain rating scale
PPT: Pressure pain threshold
SEM: Structural equation modelling
WOMAC: Western Ontario and McMaster Universities osteoarthritis index
